# The Impact of COVID-19 Quarantine Measures on Oral Hygiene Practices Among the General Public in Riyadh City, Saudi Arabia: A Cross-Sectional Study

**DOI:** 10.7759/cureus.48353

**Published:** 2023-11-06

**Authors:** Vineet Kinda, Lama Alghanim, Alanoud Aldhafayan, Sanjeev B Khanagar

**Affiliations:** 1 College of Dentistry, King Saud Bin Abdulaziz University for Health Sciences, Riyadh, SAU

**Keywords:** oral hygiene practices, quarantine, pandemic, coronavirus, covid-19, oral hygiene

## Abstract

Background and objective

Coronavirus disease 2019 (COVID-19) is an infectious disease caused by severe acute respiratory syndrome coronavirus 2 (SARS-CoV-2). It is most commonly associated with respiratory symptoms, which can vary from one individual to another. In addition, many patients can recover from this condition without undergoing any special treatment plan. In March 2020, the World Health Organization (WHO) declared COVID-19 a global pandemic and, soon after, Saudi Arabia put in place strict quarantine measures in all cities across the country to protect its citizens from the spread of COVID-19. The key objective of the quarantine program was to prevent infectious transmission from individuals who were potentially incubating the virus. Quarantine can have a significant psychological impact on human lives. With their freedom restricted and everyday life affected, people can experience behavioral changes. If people are better informed regarding such unusual situations and their effects on their oral and general health, it could go a long way in motivating them to adopt healthy practices during quarantine periods. In light of this, the aim of this cross-sectional survey was to assess the impact of quarantine on oral hygiene practices among the general public living in Riyadh City. The effect of quarantine on any changes in oral health and hygiene was also assessed.

Methodology

A cross-sectional analytical study was carried out among 570 adult participants from Riyadh, Saudi Arabia, who were aged 20 years and above. The data were collected using an online, self-administered, and structured questionnaire. The questionnaire consisted of 18 questions divided into two sections: the first part comprised questions related to demographic details and the second part consisted of questions related to oral hygiene practices and oral health.

Results

The results of the survey showed that the quarantine had affected oral hygiene practices positively by increasing the awareness of the general public about hygiene practices.

Conclusions

COVID-19 has affected our lives in various ways. Based on our findings in this study, the general population in Riyadh displayed an improvement in awareness and care about their general and oral hygiene, especially those in the age group of 20-30 years. Also, the rate of toothbrushing witnessed a positive effect during the pandemic. Some parents focused more on their children’s oral hygiene. These findings may well be attributed to the increased awareness gained through social media platforms and partly due to the fear of the potential unavailability of dental facilities during the quarantine period.

## Introduction

Coronavirus disease 2019 (COVID-19) is an infectious disease caused by severe acute respiratory syndrome coronavirus 2 (SARS-CoV-2) [1,2]. In March 2020, the World Health Organization (WHO) declared COVID-19 a global pandemic. People infected with the COVID-19 virus suffer mostly from respiratory symptoms, which can differ from one individual to another [1]. Also, many patients can recover without any special treatment plan [1]. In order to slow down the transmission of the disease or prevent it, individuals should be equipped with sufficient information about the COVID-19 virus, for instance, the nature of the disease, its causes, and the methods of transmission [1,3].

Many clinical trials have been undertaken to evaluate potential treatments for and vaccines against the rapidly emerging newer strains of the coronavirus. Since the COVID-19 pandemic is a relatively recent development, there is a scarcity of research related to its effects on oral hygiene. The majority of the existing research is about infection control related to dental clinics or about the transmission between the patient and the dentist. The American Dental Association (ADA) advised dental professionals to delay elective dental operations until April 6, 2020, and to prevent patients from blocking hospital emergency rooms, and they delivered emergency dental care only during the peak of the pandemic [4,5]. This guideline was modified on April 30, 2020, when the ADA encouraged offices to remain closed to all but very serious patients due to the spike in infections [5,6]. Some of the research has indicated that COVID-19 affects oral health due to some medications used in treating these patients [7]. 

As the transmission of COVID-19 occurred in a very rapid manner worldwide, not much is known about how to deal with this type of virus or what exactly is the nature of it [7]. In the wake of WHO declaring COVID-19 a global pandemic in March 2020, Saudi Arabia instituted quarantine measures in all cities across the country to protect its citizens from getting infected [1,8]. The key objective of the quarantine program was to prevent infectious transmission from individuals who were potentially incubating the virus [4]. Quarantine in general can have a significant psychological impact on people's lives [9,10,11,12]. Since people are locked up, their everyday lives and activities are profoundly affected. With their freedom restricted, there is a possibility of a change in their behavior [9,10,11,12]. A study conducted in Toronto found that quarantine duration was significantly associated with increased symptoms of post-traumatic stress disorder (PTSD)[9]. A large proportion of quarantined individuals suffer from anxiety, as indicated by the proportion of PTSD and depression symptoms measured using validated scales [13]. An article in the New York Times titled "The Workers Who Face the Greatest Coronavirus Risk" identified dentists as the healthcare workers who were at the highest risk of being affected by COVID-19. Dental clinics are associated with a high transmission level, especially on account of the saliva droplets from patients undergoing dental procedures [14,12,15].

To have optimal oral hygiene, the individual needs to follow the oral hygiene regime meticulously and this includes regular toothbrushing and flossing [16,17]. Regular visits to the dentist for preventive care can help with good oral hygiene maintenance. Since the dental clinics were high-risk focal points for cross-infection of COVID-19, they were shut down during the quarantine period and only provided services for emergency cases [14,18,15]. Quarantine, which was implemented in parts in various locations across the world, could affect daily oral hygiene habits among individuals in various ways [19]. The present study was conducted to analyze the attitude and practices with regard to oral hygiene among the Saudi population as well as to assess the effect of the quarantine on oral health and hygiene. This study mainly focused on addressing the following issues: do the dentists and their patients need to take more care during such quarantine periods and if any additional requirements are required on their part in terms of oral health and hygiene? If people are equipped with proper knowledge and awareness of such situations and their effects on oral and general health, it could motivate them to adopt healthy practices in similar crisis situations in the future. The aim of this cross-sectional survey was to assess the effect of quarantine on the oral hygiene practices of the general public living in Riyadh City. The effect of quarantine on any changes in oral health and hygiene was also examined.

## Materials and methods

Study design

A cross-sectional analytical study was conducted among the adult population in Riyadh City, Saudi Arabia. The research protocol was submitted to the Institutional Review Board, King Abdullah International Medical Research Center (KAIMRC), Riyadh, Saudi Arabia. KAIMRC and the College of Dentistry, King Saud Bin Abdulaziz University for Health Sciences are under the umbrella of the Ministry of National Guard Health Affairs. The data collection process was initiated after obtaining ethical clearance (Ref. no. IRBC/0428/22).

Sample size

The estimated sample size was 568, which was rounded off to 570 based on a pilot study that was done by using 5% relative precision, an expected proportion of 0.73, and a 95% confidence level [20]. The sample size was estimated using the following formula:

η=(Z^2 1-α/2)(1-p)p/℘^(2 p)

Data collection

Data were collected by using an online, self-administered, and structured questionnaire. The questionnaire consisted of 18 questions divided into two sections: the first part comprised questions about the demographic data and the second part consisted of questions related to oral hygiene practices and oral health. The questionnaire was developed by using Google Survey Forms and was distributed among the population through online portals such as WhatsApp, emails, and Twitter. Being an online survey, a convenient sampling technique was used. The study participants were informed that their privacy and confidentiality would be completely protected and that no identifiers or personal information would be collected. Written informed consent was obtained from all study participants.

Analysis was done using SPSS Statistics, Version 23 (IBM Corp., Armonk, NY). Descriptive statistics were applied to describe the distribution of the knowledge, attitude, and practices among study subjects and parameters such as gender, age, nationality, parent (yes/no), and education level. The chi-square test was applied to detect the association of different parameters with the KAP of the study population, with the statistical significance set at p<0.05.

## Results

In the present study, 570 participants responded to the survey questionnaire: 336 (58.9%) participants were female and 234 (41.1%) were male. The majority of the participants were between the age group of 20-30 years (307, 53.9%) and were Saudi nationals (555, 97.4%). Of note, 332 (58.2%) participants in this study were parents. The educational level of the participants was as follows - college level: 346 (60%), diploma/degree: 35 (6.1%), high school: 175 (30.7%), primary school: three (0.5%), and secondary school: 11 (1.9%). As shown in Table 1, most of the participants reported brushing their teeth twice daily either before or during the quarantine; however, the number of people who brushed three times a day increased from 11.0% to 18.8% during the quarantine period, which indicates increasing awareness of the hygiene practices among the general population during the pandemic.

**Table 1:** Distribution of the study population based on responses to questions related to oral hygiene practice *Mesuaq is a toothbrush made from Salvadora tree

Responses to questions related to oral hygiene practice			
		N	%
How many times did you brush your teeth before the COVID-19 quarantine?	3 times a day	63	11.1
	Never	23	4
	Once daily	223	39.1
	Twice daily	261	45.8
How many times did you brush your teeth during the COVID-19 quarantine?	3 times a day	107	18.8
	Never	22	3.9
	Once daily	193	33.9
	Twice daily	248	43.5
How many times did you use Mesuaq* before the COVID-19 quarantine?	3 times a day	1	0.2
	Daily	30	5.3
	Monthly	96	16.8
	Never	401	70.4
	Once daily	1	0.2
	Twice daily	2	0.4
	Weekly	39	6.8
How many times did you use Mesuaq during the COVID-19 quarantine?	3 times a day	2	0.4
	Daily	26	4.6
	Monthly	58	10.2
	Never	437	76.7
	Once daily	1	0.2
	Twice daily	1	0.2
	Weekly	45	7.9
Did you take more care of your oral hygiene during your free time during the COVID-19 quarantine?	No	315	55.3
	Yes	255	44.7

As shown in Table 2, regarding oral hygiene practice ("How many times did you brush your teeth before/after the COVID-19 pandemic?"), the Wilcoxon signed-rank test indicated the population describing a higher rate of toothbrushing during COVID-19 quarantine (p=0.001).

**Table 2:** Comparison of oral hygiene practices during and before COVID-19 quarantine *Statistically significant

			N	Mean rank	P-value
How many times did you brush your teeth?	During COVID-19 quarantine – Before COVID-19 quarantine	Negative ranks	29	70.55	0.001*
		Positive ranks	94	59.36	
		Ties	447		
How many times did you use Mesuaq?	During COVID-19 quarantine – Before COVID-19 quarantine	Negative ranks	22	38.95	0.001*
		Positive ranks	54	38.31	
		Ties	494		

Similarly, statistical analysis showed that regarding oral hygiene practice, in terms of the participants' frequency of using Mesuaq before/during the COVID-19 quarantine, the population used Mesuaq more frequently during the COVID-19 quarantine (p=0.001); 27.5% of the parents had focused more on their children’s oral hygiene while 15.4% did not (Table 3). Only about 20% of the participants had a dental problem (pain) during the quarantine and about 43% of participants who had dental problems did not do anything about it. Only 24.2% of the respondents stated that quarantine had an influence on their oral hygiene, which shows that perhaps the population studied already had satisfactory oral hygiene practices. Only 21.9% of the participants thought that the closing of dental clinics had an effect on their oral hygiene.

**Table 3:** Distribution of responses to questions on quarantine-related oral hygiene and dental problems

Responses to questions on quarantine-related oral hygiene and dental problems			
		N	%
If you’re a parent, did you focus more on your children’s hygiene during quarantine?	No	88	15.4
	Yes	157	27.5
	Not a parent	325	57
Did you have a dental problem during quarantine?	Yes	114	20
	No	456	80
If yes, answer the following question: what did you do to solve this problem?	Asked relatives/friends	14	12.3
	Consulted a specialist online	27	23.7
	Looked for solutions online	11	9.6
	Nothing	49	43
	Used traditional treatment	13	11.4
Did the quarantine have an effect on your oral hygiene routine?	Decreased	41	7.2
	Don’t know	79	13.9
	Increased	138	24.2
	No effect	312	54.7
Did the closing of the dental clinics during quarantine affect/change your oral hygiene?	No	445	78.1
	Yes	125	21.9

As shown in Table 4, three questions were combined and analyzed to determine the association between responses. Chi-Square analysis displayed no statistically significant difference in the responses to the question “Did you take more care of your oral hygiene during your free time in COVID-19 quarantine?” among participants of different genders, age groups, and education levels (p>0.05). However, the Chi-Square analysis displayed a statistically significant association among participants from different nationalities and parenthood. Most of the Saudi nationals and non-Saudi nationals responded that the closure of the dental clinics during quarantine did not have any effect/change on their oral hygiene. Similarly, most parents and non-parents responded “No” to the question “Did the closure of the dental clinics during quarantine affect/change your oral hygiene?” (p<0.05). Chi-Square analysis displayed no statistically significant difference in the response to the question “Did you have a dental problem during quarantine?” among participants of different genders, age groups, and education levels (p>0.05). However, Chi-Square analysis displayed a statistically significant association among different age groups, parenthood, and education levels with regard to the response to the question “Did the quarantine have an effect on your oral hygiene routine?” The age group of 20-30 years displayed an “Increased” effect on oral hygiene routine whereas all the other groups showed that there was “No effect” on oral hygiene routine. Similarly, participants of all educational levels and different parenthoods chose “No effect” as the common answer to the question on the effect of quarantine on oral hygiene routine (p<0.05).

**Table 4:** Association among different groups of participants regarding responses to questions

Association among different groups of participants						
		Question	No	Yes	Chi-square value	P-value
Gender	Female	Did you take more care of your oral hygiene during your free time in the COVID-19 quarantine?	54.20%	45.80%	0.398	0.528
			56.89%	43.20%		
	Male					
		Did the closing of the dental clinics during quarantine affect/change your oral hygiene?	77.40%	22.60%	0.227	0.634
			79.10%	20.90%		
		Did you have a dental problem during quarantine?	58.10%	62.30%	0.654	0.419
			41.90%	37.70%		
Age group, years	20-30	Did you take more care of your oral hygiene during your free time in the COVID-19 quarantine?	54.30%	53.30%	2.72	0.436
	31-45		27.90%	32.90%		
	46-60		14.30%	11.40%		
			3.50%	2.40%		
							Over 60	Did the closing of the dental clinics during quarantine affect/change your oral hygiene?	55.70%	47.20%	6.11	0.106
	30.10%	30.40%										
	11.20%	19.20%										
			2.90%	3.20%								
	Did you have a dental problem during quarantine?	52.40%	59.60%	7.82	0.05							
		32.50%	21.10%				
		11.80%	17.50%				
		3.30%	1.80%				
	Educational level	College	Did you take more care of your oral hygiene during your free time in the COVID-19 quarantine?	54.00%	46.00%	3.34		0.502
		Diploma/degree		57.10%	42.90%			
58.90%				41.10%				
High school		33.30%		66.70%				
		36.40%		63.60%				
Primary school		Did the closing of the dental clinics during quarantine affect/change your oral hygiene?	78.30%	21.70%	3.2	0.511
			71.40%	28.60%		
			79.40%	20.60%		
Secondary school						
			100%	0%		
			63.60%	36.40%		
		Did you have a dental problem during quarantine?	61.00%	59.60%	2.65	0.618
			5.50%	8.80%		
			31.40%	28.10%		
			0.40%	0 .90%		
			1.80%	2.60%		

## Discussion

COVID-19 emerged in China toward the end of 2019 and quickly spread to other parts of the world. It is caused by SARS-CoV-2, which affects the respiratory system. Because of its rapid and easy spread, it has become a pandemic in almost every country, forcing governments to impose quarantine measures to slow down the spread of the virus and reduce the number of deaths. However, such practices may lead to a variety of side effects on people, one of the most significant of which pertains to oral health care. Since dental clinics are associated with a high transmission level of pathogens, they were closed during the peak of the pandemic. The aim of this cross-sectional survey was to study the effect of quarantine on oral hygiene practices among the general public living in Riyadh City. The effect of quarantine on changes in oral health and hygiene was also studied. 

A systemic review focusing on the influence of the COVID-19 pandemic on oral hygiene, dietary habits, and caries disease among adults reported that patients with halitosis were more conscious of their poor breath due to their regular mask use, which led to increased toothbrushing frequency [2]. On the other hand, some people reduced the frequency of their brushing and showed less care about the appearance of their teeth because their faces were always covered with a mask [2]. Therefore, masks were one of the factors that led to both an increase in caries risk in one group of responders as well as a reduction in caries risk in another group [21]. A study conducted in Saudi Arabia involving 211 individuals assessed the impact of COVID-19 on personal hygiene [21]. The authors reported that the participants' personal hygiene practices showed improvement at various degrees as compared to the pre-pandemic period [21]. They concluded that the pandemic led to a considerable improvement in personal hygiene behaviors in Saudi Arabia, particularly those linked to COVID-19 prevention [21]. Our study, which focused more on oral hygiene, had similar findings. It indicated that the Saudi population is aware of personal hygiene and oral hygiene in particular.

Another study discussed whether COVID-19 has improved public awareness based on information available on Google Trends [22]. It found that following the outbreak of the COVID-19 epidemic, the general public appeared to be more concerned about their dental hygiene, which aligns with our findings [22]. A study conducted in the United States targeted adolescents and young adults to examine their perspectives on dental health and oral hygiene practices [23]; 44% of the survey participants stated that COVID-19 affected their oral health [23]. Also, they reported that people who had COVID faced scheduling issues and worsening oral hygiene [23]. A similar study was conducted to evaluate and compare how the COVID-19 quarantine has affected Portuguese and Spanish children's attitudes toward oral health, dietary practices, and availability of dental treatment [24]. It reported that just 12.9% of Spanish children and 14.3% of Portuguese children increased the frequency of brushing their teeth during quarantine compared to the pre-pandemic period [24], whereas our survey showed that 27.5% of parents had focused more on their children’s oral hygiene [24].

Another study involving a group of Chinese children on oral hygiene practices during the quarantine revealed that 84.2% of them did not alter their brushing frequency [25]. Our data showed a higher rate of toothbrushing among the study participants; however, a cross-sectional study to assess oral habits during the COVID-19 lockdown in Romania [26] found that there was no difference in the frequency of toothbrushing among the study participants [26], whereas the medical and dental group had an increase in the amount of time spent brushing during the quarantine compared to before, with more individuals brushing for at least two minutes [26]. The present study has a few limitations, especially since the data was collected through online portals due to COVID-19 restrictions. Another limitation was the relative scarcity of literature in terms of other comparable studies with similar objectives related to oral hygiene and COVID-19.

## Conclusions

The COVID-19 pandemic has affected our lives in various ways. Based on the findings of the present study, the general population in Riyadh displayed an improvement in awareness and care about their general and oral hygiene, especially those in the age group of 20-30 years. Also, the rate of brushing was affected positively during the pandemic. Some parents focused more on their children’s oral hygiene. These findings may well be attributed to increased awareness among the participants through social media platforms and partly due to the fear of the potential unavailability of dental facilities during the pandemic. Further research into these aspects could throw up interesting results.
